# Long‐term survival of a patient with epidermal growth factor receptor (EGFR)‐mutant non‐small cell lung cancer (NSCLC) and untreated multiple brain metastases treated with zorifertinib: A case report

**DOI:** 10.1111/1759-7714.15317

**Published:** 2024-04-26

**Authors:** Kang Li, Bolin Chen, Jingyi Wang, Lin Wu

**Affiliations:** ^1^ Department of Thoracic Medical Oncology Hunan Cancer Hospital/The Affiliated Cancer Hospital of Xiangya School of Medicine, Central South University Changsha China

**Keywords:** brain metastases, *EGFR*‐mutant NSCLC, third‐generation EGFR‐TKI, zorifertinib

## Abstract

Brain metastases (BM) are common in patients with epidermal growth factor receptor (EGFR)‐mutant non‐small cell lung cancer (NSCLC) and confer poor prognoses. Zorifertinib (AZD3759), an EGFR‐tyrosine kinase inhibitor (TKI) with high blood‐brain barrier penetration, has previously demonstrated promising systemic and intracranial antitumor activity in phase 1–3 studies. This is the first report of a patient with *EGFR*‐mutant (exon 21 L858R) NSCLC and symptomatic untreated multiple BM who achieved a long overall survival (OS) of more than 65 months after sequential treatment with zorifertinib and a third‐generation EGFR‐TKI. This new treatment paradigm offers a new treatment option and deserves further clinical exploration to prolong OS of patients with *EGFR*‐mutant NSCLC and untreated multiple BM.

## Graphical abstract

Long OS achieved in NSCLC patients with EGFR mutation and untreated multiple BM after zorifertinib treatment.

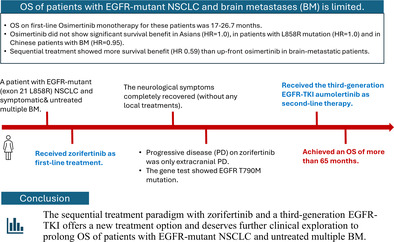

## INTRODUCTION

Brain metastases (BM) in patients with epidermal growth factor receptor (*EGFR*)‐mutant non‐small cell lung cancer (NSCLC) are very common and confer poor prognoses.^1^ EGFR tyrosine kinase inhibitors (EGFR‐TKIs) are the systemic therapy recommended by most treatment guidelines for patients with *EGFR*‐mutant NSCLC and BM.^1^, ^2^, ^3^, ^4^, ^5^ Overall survival (OS) on first‐line osimertinib monotherapy for these patients has previously been reported to be 17–26.7 months.^6^, ^7^, ^8^, ^9^

Zorifertinib (AZD3759), a novel EGFR‐TKI inhibiting mutant *EGFR* (exon 19del or L858R), is not a substrate of either P‐glycoprotein or breast cancer resistance protein and was designed specifically to improve blood–brain barrier (BBB) penetration^10^, ^11^ and to treat *EGFR*‐mutant NSCLC patients with BM. In phase 1–3 studies, zorifertinib has shown promising antitumor activity.^10^, ^12^, ^13^

This is the first report of a patient with *EGFR*‐mutant (exon 21 L858R) NSCLC and symptomatic untreated multiple BM who achieved a long OS after sequential treatment with zorifertinib and aumolertinib.

## CASE REPORT

**FIGURE 1:**
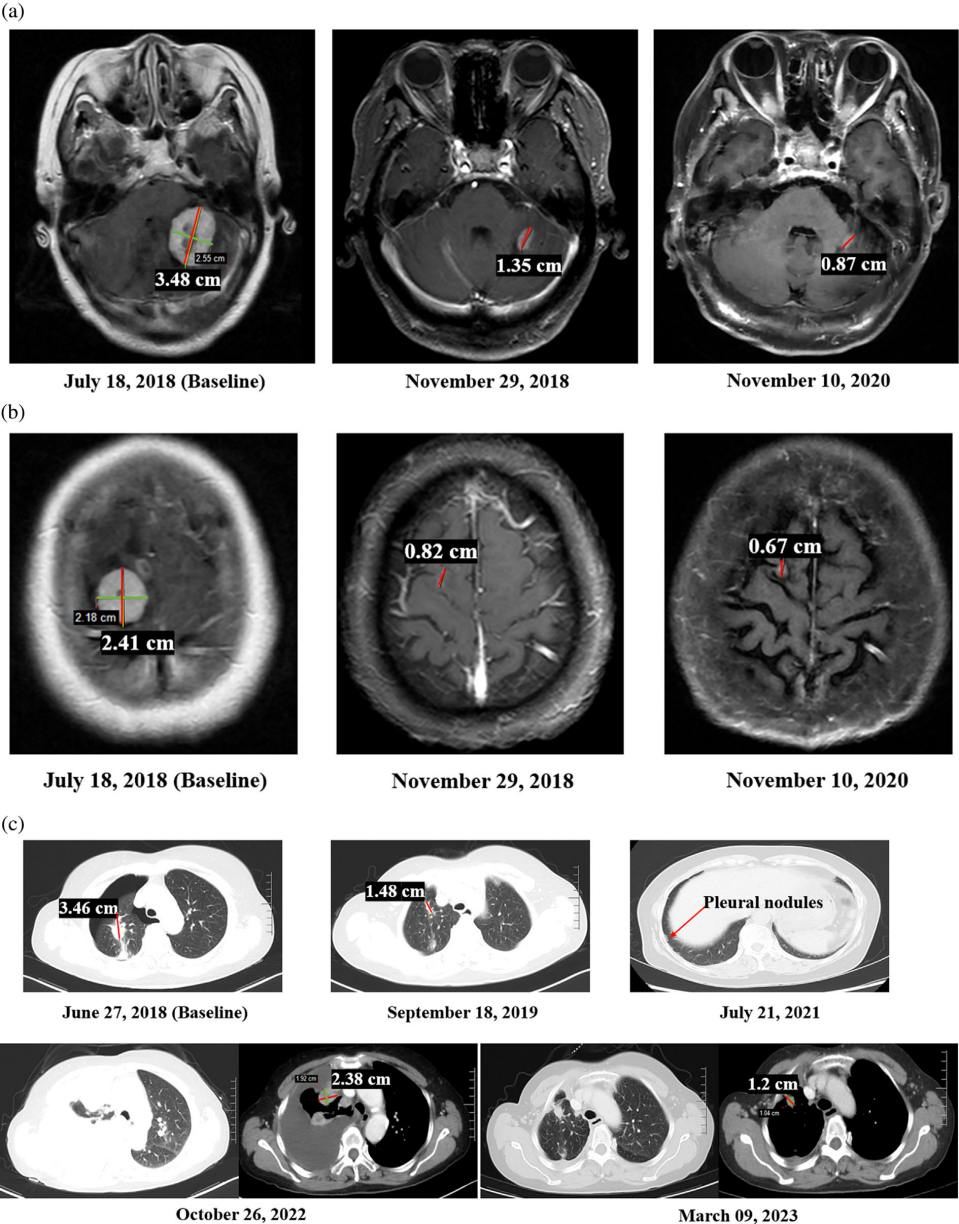
Image screenshots of the three target lesions. (a) Brain magnetic resonance images of the No. 1 target lesion in the left cerebellar hemisphere: it was 34.8 mm in diameter on July 18, 2018 (baseline), 1.35 mm in diameter on November 29, 2018, and 0.87 mm in diameter on November 10, 2020. (b) Brain magnetic resonance images of No. 2 target lesion in the right cerebral hemisphere: it was 24.1 mm in diameter on July 18, 2018 (baseline), 0.82 mm in diameter on November 29, 2018, and 0.67 mm in diameter on November 10, 2020. (c) Thoracic computed tomography scans of No. 3 target lesion in the right upper lung: it was 34.6 mm in diameter on June 27, 2018 (baseline) and 14.8 mm in diameter on September 18, 2019. On July 21, 2021, pleural nodules appeared. On October 26, 2022, the chest CT showed massive pleural effusion and significant progressive disease of the pleural nodules. On March 09, 2023, the pleural effusion disappeared, and the pleural nodules had shrunk significantly.

A 65‐year‐old Asian, nonsmoking female was initially diagnosed with *EGFR*‐mutant (exon 21 L858R) NSCLC and multiple BM (adenocarcinoma; T2aN0M1c, stage IVb) in June 2018. She was enrolled in a phase 1–2 clinical trial which evaluated the safety, tolerability, pharmacokinetics, and antitumor activity of zorifertinib in Chinese patients with *EGFR*‐mutant NSCLC and BM (NCT03360929) on July 12, 2018. There were three target lesions (24.1–34.8 mm, Figure 1) with a total diameter of 93.5 mm, and multiple brain nontarget lesions at baseline. The ECOG performance status was 1. She had neurological symptoms at baseline including gait abnormalities (abnormal but walks without assistance) and decreased strength (movement present but decreased against resistance).

**FIGURE 2:**
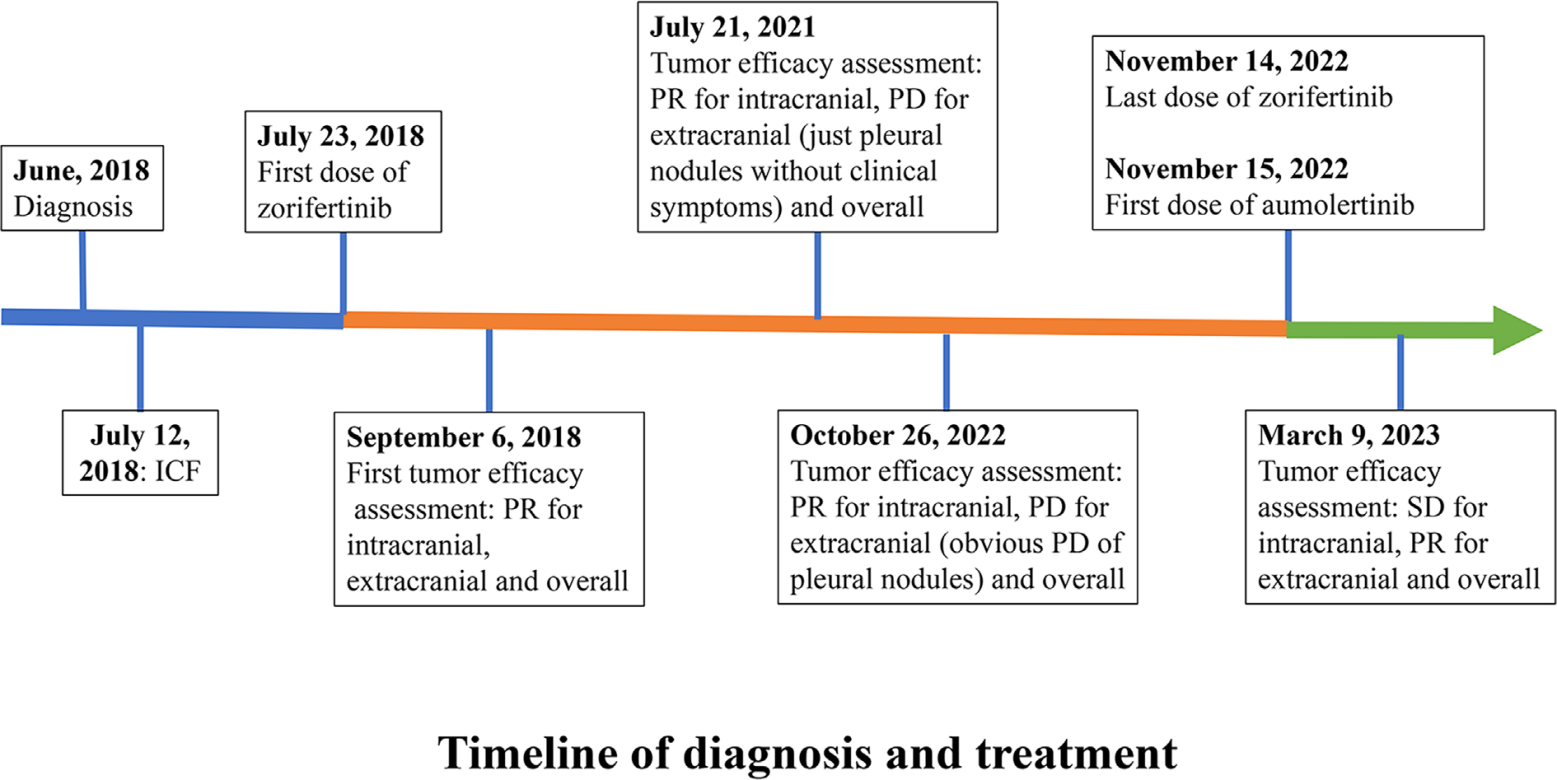
The whole timeline of diagnosis and treatment of this patient. ICF, informed consent form; PD, progressive disease; PR, partial response; SD, stable disease.

The patient received zorifertinib as first‐line treatment administered orally for 21‐day cycles starting on July 23, 2018. The starting dose of zorifertinib was 250 mg twice daily, and finally maintained at 150 mg twice daily after two dose reductions (Table 1). She continued on zorifertinib as she had shown clinical benefit (only single‐site metastasis without related symptoms, the intracranial lesions were still responding) when the first progressive disease (PD) in just the pleural nodules occurred on July 21, 2021. On October 26, 2022, the pleural nodules significantly progressed and the gene test using the pleural effusion showed EGFR T790M mutation. She was then prescribed the marketed third‐generation EGFR‐TKI aumolertinib as second‐line therapy. For a timeline of diagnosis to treatment, please refer to Figure 2.

**TABLE 1:** Dosage of zorifertinib and dose reductions^a^.

Content	The starting dose	Dose after first dose reduction	Dose after second dose reduction
Dose level	250 mg BID	200 mg BID	150 mg BID
Reasons for dose reduction	Not applicable	Dermatitis acneiform of grade 3	Increased aspartate aminotransferase of grade 3
Starting date	July 23, 2018	September 17, 2018	January 16, 2019

Abbreviation: BID, twice daily.

^a^ Per the requirement of the NCT03360929 study protocol, dose reduction was allowed when a treatment‐related grade 3 advert event occurs.

The radiographic assessments per response evaluation criteria in solid tumors version 1.1 (RECIST1.1) following zorifertinib treatment were partial response (PR) for all the intra‐, extracranial, and overall tumor responses (Figure 1). The patient's neurological symptoms gradually improved and completely disappeared on October 18, 2018 after four‐cycle zorifertinib therapy (without any local treatments [requirement of the NCT03360929 study protocol]); and there were no relapses thereafter. Progression‐free survival (PFS) of first‐line zorifertinib treatment was 3 years, while the intracranial PFS was more than 4 years. By December 25, 2023, the duration of second‐line aumolertinib therapy was 13 months. The patient was still alive, and the OS was more than 65 months since the beginning of treatment with zorifertinib.

Most treatment‐related adverse events (AEs) with zorifertinib were grade 1–2 (Table 2). Only dermatitis acneiform and aspartate aminotransferase increase were grade 3; both recovered after dose reduction (Table 1) and symptomatic treatment (S1 data, Appendix Table S1).

**TABLE 2:** All treatment‐related adverse events summarized by maximum severity^a^ when the patient was taking zorifertinib.

Grade 1	Grade 2	Grade 3
1. Abdominal discomfort	1. Blood bilirubin increase	1. Aspartate aminotransferase increase
2. Alanine aminotransferase increase	2. Conjunctivitis	2. Dermatitis acneiform
3. Blood urea increase	3. Paronychia	
4. Diarrhea	4. Stomatitis	
5. Leukocytosis		
6. Neutrophil count increase		
7. Proteinuria		
8. Rhinorrhea		
9. Vomiting		
10. Weight increase		

^a^ Adverse events were graded for severity using the National Cancer Institute Common Terminology Criteria for Adverse Events version 4.03 (CTCAE 4.03).

## DISCUSSION

The third‐generation EGFR‐TKI osimertinib has not previously shown significant survival benefit in Asians (HR = 1.0, 95% confidence interval [CI]: 0.75–1.32), in patients with L858R mutation (HR = 1.0, 95% CI: 0.71–1.40) and in Chinese patients with BM (HR = 0.95, 95% CI: 0.45–1.97) in FLAURA and FLAURA China studies.^14^, ^15^ In four retrospective studies, OS following first‐line osimertinib monotherapy for brain‐metastatic patients was less than 30 months.^6^, ^7^, ^8^, ^9^ In the phase 2 APPLE trial, the sequential treatment showed more survival benefit (HR = 0.59) than upfront osimertinib in brain‐metastatic patients.^16^ Additionally, the mechanism of acquired resistance to third‐generation EGFR‐TKIs is very complex; optimizing the next step after PD is an ongoing challenge.^17^

Zorifertinib demonstrated 100% BBB penetration, which is much higher than that of other EGFR‐TKIs (first‐ and second‐generation EGFR‐TKIs, 1.13% to 3.3%; osimertinib, 2.5% to 16%).^10^, ^18^ In previous studies, first‐line zorifertinib was particularly effective in controlling BM (extracranial PD is the main pattern of PD), and the main acquired resistance mutation was EGFR T790M which is subsequently treatable with third‐generation EGFR‐TKIs, helping to prolong OS of patients; the median OS was 34.1 months in patients subsequently treated with osimertinib in CTONG1702, and is consistent with that of the phase 3 EVEREST study (data on file).^12^, ^13^

Different from other case reports of long OS in *EGFR*‐mutant NSCLC patients with initially asymptomatic, solitary BM or initially no BM after four‐line therapies including combination with radiotherapy, or off‐label use of osimertinib (double dose),^19^, ^20^ the patient in this report had more poor prognostic factors, including EGFR L858R and initially diagnosed symptomatic multiple BM. She also received only sequential treatment of zorifertinib and a third‐generation EGFR‐TKI without intracranial radiotherapy. Her neurological symptoms completely recovered. She achieved PR in both extra‐ and intracranial tumor lesions, developed EGFR T790M mutation after PD on zorifertinib, and finally achieved an OS of more than 65 months. The AEs with zorifertinib were consistent with those reported in phase 1–3 studies and other EGFR‐TKIs.^10^, ^12^, ^13^

These results demonstrate that sequential treatment of zorifertinib and third‐generation EGFR‐TKIs may be a better treatment option for patients with *EGFR*‐mutant NSCLC and untreated multiple BM, as zorifertinib is a high BBB penetrant and well‐validated EGFR‐TKI with T790M as the main resistant mutation, which is treatable with the third‐generation EGFR‐TKIs. This sequential treatment offers a new treatment option and deserves further clinical exploration to prolong the OS of these patients.

## AUTHOR CONTRIBUTIONS

All authors had full access to the data in the study and take responsibility for the integrity of the data and accuracy of the data analysis. Conceptualization: Kang Li and Lin Wu. Writing—original draft preparation. Kang Li and Bolin Chen. Visualization: Kang Li and Jingyi Wang. Writing—review and editing: All authors.

## CONFLICT OF INTEREST STATEMENT

The authors have no conflicts of interest to declare.

## Supporting information


**Table S1.** Symptomatic treatments for dermatitis acneiform and aspartate aminotransferase increased of grade 3.

## Data Availability

The original contributions presented in the report are included in the article. Further enquiries can be directed to the corresponding author.

## References

[1] Vogelbaum MA , Brown PD , Messersmith H , Brastianos PK , Burri S , Cahill D , et al. Treatment for brain metastases: ASCO‐SNO‐ASTRO guideline. J Clin Oncol. 2022;40(5):492–516. 34932393 10.1200/JCO.21.02314

[2] National Comprehensive Cancer Network: NCCN clinical practice guidelines in oncology (NCCN guidelines®) non‐small cell lung cancer version 2.2024. 2024 https://www.nccn.org/professionals/physician_gls/pdf/nscl.pdf

[3] Oncology Society of Chinese Medical Association, Chinese Medical Association Publishing House . Chinese Medical Association guideline for clinical diagnosis and treatment of lung cancer (2022 edition). Zhonghua Zhong Liu Za Zhi. 2022;44(6):457–490. 35754224 10.3760/cma.j.cn112152-20220413-00255

[4] Chinese Association for Clinical Oncologists, Medical Oncology Branch of Chinese International Exchange and Promotion Association for Medical and Healthcare . Clinical practice guideline for brain metastases of lung cancer in China (2021 version). Zhonghua Zhong Liu Za Zhi. 2021;43(3):269–281. 33752305 10.3760/cma.j.cn112152-20210104-00009

[5] Passaro A , Leighl N , Blackhall F , Popat S , Kerr K , Ahn MJ , et al. ESMO expert consensus statements on the management of EGFR mutant non‐small‐cell lung cancer. Ann Oncol. 2022;33(5):466–487. 35176458 10.1016/j.annonc.2022.02.003

[6] Zhai X , Li W , Li J , Jia W , Jing W , Tian Y , et al. Therapeutic effect of osimertinib plus cranial radiotherapy compared to osimertinib alone in NSCLC patients with EGFR‐activating mutations and brain metastases: a retrospective study. Radiat Oncol. 2021;16(1):233. 34865626 10.1186/s13014-021-01955-7 PMC8647301

[7] Yu F , Ni J , Zeng W , Zhou Y , Guo T , Zeng Y , et al. Clinical value of upfront cranial radiation therapy in osimertinib‐treated epidermal growth factor receptor‐mutant non‐small cell lung cancer with brain metastases. Int J Radiat Oncol Biol Phys. 2021;111(3):804–815. 34058255 10.1016/j.ijrobp.2021.05.125

[8] Zhao Y , Li S , Yang X , Chu L , Wang S , Tong T , et al. Overall survival benefit of osimertinib and clinical value of upfront cranial local therapy in untreated EGFR‐mutant non‐small cell lung cancer with brain metastasis. Int J Cancer. 2022;150(8):1318–1328. 34914096 10.1002/ijc.33904

[9] Zhang BY , He ZL , Wu S . Prognostic analysis of osimertinib in the treatment of brain metastases from EGFR‐mutant non‐small‐cell lung cancer. Tumor. 2021;41(02):110–120.

[10] Ahn MJ , Kim DW , Cho BC , Kim SW , Lee JS , Ahn JS , et al. Activity and safety of AZD3759 in EGFR‐mutant non‐small‐cell lung cancer with CNS metastases (BLOOM): a phase 1, open‐label, dose‐escalation and dose‐expansion study. Lancet Respir Med. 2017;5(11):891–902. 29056570 10.1016/S2213-2600(17)30378-8

[11] Yang Z , Guo Q , Wang Y , Chen K , Zhang L , Cheng Z , et al. AZD3759, a BBB‐penetrating EGFR inhibitor for the treatment of EGFR mutant NSCLC with CNS metastases. Sci Transl Med. 2016;8(368):ra172. 10.1126/scitranslmed.aag0976 27928026

[12] Maggie Liu SY , Dong XR , Wang Z , du Y , Cui JW , Chu Q , et al. Efficacy, safety and dose selection of AZD3759 in patients with untreated EGFR‐mutated non‐small‐cell lung cancer and central nervous system metastases in China (CTONG1702‐arm 8): a multi‐center, single‐arm, phase 2 trial. EClinicalMedicine. 2023;64:102238. 37781161 10.1016/j.eclinm.2023.102238 PMC10541475

[13] Wu Y‐L , Zhou Q , Wang J , Yu Y , Xing L , Wang Y , et al. Randomized phase 3 study of first‐line AZD3759 (zorifertinib) versus gefitinib or erlotinib in EGFR‐mutant (EGFR m+) non‐small‐cell lung cancer (NSCLC) with central nervous system (CNS) metastasis. J Clin Oncol. 2023;41(16_suppl):abstr9001.

[14] Ramalingam SS , Vansteenkiste J , Planchard D , Cho BC , Gray JE , Ohe Y , et al. Overall survival with Osimertinib in untreated, EGFR‐mutated advanced NSCLC. N Engl J Med. 2020;382(1):41–50. 31751012 10.1056/NEJMoa1913662

[15] Cheng Y , He Y , Li W , Zhang HL , Zhou Q , Wang B , et al. Osimertinib versus comparator EGFR TKI as first‐line treatment for EGFR‐mutated advanced NSCLC: FLAURA China: a randomized study. Target Oncol. 2021;16(2):165–176. 33544337 10.1007/s11523-021-00794-6 PMC7935816

[16] Remon J , Besse B , Aix SP , Callejo A , al‐Rabi K , Bernabe R , et al. Overall survival from the EORTC LCG‐1613 APPLE trial of osimertinib versus gefitinib followed by osimertinib in advanced *EGFR*‐mutant non‐small‐cell lung cancer. J Clin Oncol. 2024;42(12):1350–1356. 38324744 10.1200/JCO.23.01521

[17] Gomatou G , Syrigos N , Kotteas E . Osimertinib resistance: molecular mechanisms and emerging treatment options. Cancers (Basel). 2023;15(3):841. 36765799 10.3390/cancers15030841 PMC9913144

[18] Cheng H , Perez‐Soler R . Leptomeningeal metastases in non‐small‐cell lung cancer. Lancet Oncol. 2018;19:e43–e55. 29304362 10.1016/S1470-2045(17)30689-7

[19] Wan Y , Xu F , Wang J . Long‐term survival of a non‐small cell lung cancer patient with EGFR‐mutated brain metastases: a case report. Transl Cancer Res. 2022;11(12):4448–4454. 36644180 10.21037/tcr-22-1671 PMC9834592

[20] Sun Y , Ai X , Lu S . Tagrisso incremental therapy in a case of meningeal metastasis of lung cancer with EGFR mutation: a case report. Transl Lung Cancer Res. 2022;11(2):323–330. 35280312 10.21037/tlcr-21-451 PMC8902093

