# Supplementary material for: Long‐term survival of a patient with epidermal growth factor receptor (EGFR)‐mutant non‐small cell lung cancer (NSCLC) and untreated multiple brain metastases treated with zorifertinib: A case report
Source: Thorac Cancer. 2024 Apr 26;15(16):1325–9. doi: 10.1111/1759-7714.15317 (PMC11147662; doi:10.1111/1759-7714.15317)
Supplement: Supplementary file 1 — Table S1. Symptomatic treatments for dermatitis acneiform and aspartate aminotransferase increased of grade 3. [file TCA-15-1325-s001.doc]

# APPENDIX

**Long-term survival of a patient with EGFR-mutant NSCLC and untreated multiple brain metastases treated with zorifertinib: A case report**

Kang LI, Bolin CHEN, Jingyi WANG, Lin WU

Table S1 Symptomatic treatments for dermatitis acneiform and aspartate aminotransferase increased of grade 3.

|  | Dermatitis acneiform of grade 3 | Increased aspartate aminotransferase of grade 3 |
| --- | --- | --- |
| Oral medication | Methylprednisolone Tablets | Diammonium glycyrrhizinate capsules; Bifendate pills |
| Intravenous medications | Methylprednisolone sodium succinate for injection | Magnesium isoglycyrrhizinate injection; Reduced glutathione for injection |
| Topical medications | Triamcinolone acetonide and econazole nitrate cream; Mometasone furoate gel; Fusidic acid cream; Desonide cream; Aloe vera gel | None |
